# *Leioproctus rosellae* sp. n., the first record of the genus from northern South America (Hymenoptera, Colletidae)

**DOI:** 10.3897/zookeys.141.2029

**Published:** 2011-10-28

**Authors:** Victor H. Gonzalez, Jaime Florez

**Affiliations:** 1 Division of Entomology, Natural History Museum, 1501 Crestline Drive – Suite 140, University of Kansas, Lawrence, Kansas 66045, USA; 2 Department of Biology, Utah State University, Logan, Utah, 84322-5310, USA; 3 Fundación Nativa para la Conservación de la Diversidad, Cali, Colombia

**Keywords:** Anthophila, Apoidea, *Bicolletes*, Paracolletini, taxonomy

## Abstract

*Leioproctus* Smith is a diverse colletine genus found in the Australian region and primarily temperate areas of South America. A new species of *Leioproctus* subgenus *Perditomorpha* Ashmead, *Leioproctus rosellae* Gonzalez, **sp. n.**, from a tropical dry forest of the Caribbean coast of Colombia is described and figured. This is the first record of the genus from northern South America.

## Introduction

The purpose of this paper is to describe a new species of the Paracolletini genus *Leioproctus* Smith (*sensu* Michener 2007) from a tropical dry forest of the Caribbean coast of Colombia. *Leioproctus* is a species-rich genus found in the Australian region and primarily temperate areas of South America. It consists of more than 300 species grouped into 35 subgenera (Michener 2007) that are treated at the generic level by some authors (e.g., Almeida 2008; Moure et al. 2008; Ascher and Pickering 2011). In South America, species of the 18 subgenera of *Leioproctus* are currently known from Chile and Argentina to central Peru and northeastern Brazil (Michener 2007; Moure et al. 2008; Ascher and Pickering 2011); thus, this Colombian record considerably extends the distribution of the genus in the Western Hemisphere.

*Leioproctus* s.l. is “a practical solution to an uncomfortable problem”, as stated simply by Michener (2007) [quotations added]. The genus has long been suspected to be paraphyletic (Michener 1989, 2007), and a recent higher level phylogenetic analysis of Colletidae using molecular data supports this view (Almeida and Danforth 2009). It may seem straightforward to treat all subgenera of *Leioproctus* s.l. at the generic level to solve this problem, as adopted by other authors and suggested by Almeida and Danforth (2009), but work still remains to develop a stable, informative generic and subgeneric classification. Some authors (e.g., Silveira et al. 2002; Moure et al. 2008) not only recognize at the generic level all South American subgenera of *Leioproctus sensu* Michener (2007), but also those unusual species or species groups synonymized by him (e.g., *Belopria* Moure, *Edwyniana* Moure). The recognition of such unusual species or species groups at the generic level seems an unnecessary splitting that conveys little information regarding phylogenetic relationships. Others authors (i.e., Ascher and Pickering 2011), recognize only some of Michener’s subgenera at the generic level while synonymizing others. This is the case for *Perditomorpha* Ashmead, the largest subgenus of *Leioproctus* in South America containing more than 40 described species. Michener (1989) synonymized *Bicolletes* Friese, *Edwyniana*, and *Belopria* with *Perditomorpha* given the morphological variation among species and the existence of taxa with intermediate morphologies among groups. However, Ascher and Pickering (2011) treat *Perditomorpha* at the generic level, with the inclusion of *Bicolletes* Friese, *Perditomorpha* s. str. and *Kylopasiphae* Michener as only subgenera; *Kylopasiphae* is included here even though it is considered a separated subgenus by (Michener (1989, 2007) or genus by Moure et al. (2008).

The species described herein belongs to *Perditomorpha* (*sensu* Michener 2007), a taxon currently known from both sides of the Chilean and Argentinean Andes, north to Peru and Bolivia, and the state of Ceará, Brazil (Michener 2007). The Colombian species is allied to the *neotropicus* species group, an assemblage containing the majority of *Perditomorpha* species that are or have been placed in the genus or subgenus *Bicolletes*. Thus, depending of the classification followed, the new species could be described as a member of *Leioproctus*, *Perditomorpha* or *Bicolletes*. Until a comprehensive morphological phylogenetic study is done to help us evaluate the relative merits of recognizing separate genera within this group of colletines, we have conservatively decided to follow Michener’s generic and subgeneric classification of *Leioproctus*.

The presence of this new species in the dry forests of the Colombian Caribbean is interesting but not surprising given that bees from this region are poorly collected and underrepresented in collections. Tropical dry forests in Colombia are primarily found along the Caribbean coast and the valleys of the Magdalena and Cauca rivers (Espinal and Montenegro 1977). These forests are known to contain not only several endemic species [e.g., *Acamptopoeum colombiensis* Shinn] but, recently, also taxa previously unknown from South America or restricted to southern South America. For example, the osmiine genus *Heriades* Spinola (Megachilidae), a taxon previously known from North and Central America (Gonzalez and Griswold, personal observations), and the oil-collecting genus *Tapinotaspoides* Moure (Apidae, Tapinotaspidini), previously known from Argentina and Paraguay and southeastern Brazil, have been recently collected in these coastal forests (Gonzalez and Ospina 2006; Melo and Aguiar 2008). Such records, as well as the species described here, suggest the existence of an interesting bee fauna that deserves to be thoroughly explored.

## Material and methods

Morphological terminology follows that of (Engel (2001, 2009) and Michener (2007). As in Engel (2009), the projections from the inner metatibial spur are herein called branches, instead of teeth. Measurements were taken using an ocular micrometer on a Leica® MZ12 stereomicroscope. Photomicrographs were taken using a Keyence® VHX-500F Digital Imaging System.

## Systematics

### Leioproctus (Perditomorpha) rosellae

Gonzalez sp. n.

urn:lsid:zoobank.org:act:093B2989-D44D-4F90-B245-9693AB38335F

http://species-id.net/wiki/Leioproctus_(Perditomorpha)_rosellae

Figs 1–5 

#### Holotype.

 ♀, Colombia: Magdalena, Santa Marta, via a Nenguange, cerca a Bonda [11°24'N, 74°12'W], Enero 3, 2007, J. Florez. Deposited in the Instituto de Ciencias Naturales, Universidad Nacional de Colombia, Bogotá, Colombia.

#### Diagnosis.

 The female of this species belongs to the *neotropicus* species group *sensu* Michener (2007) mainly distinguished by the coarsely pectinate inner metatibial spur and weakly developed sternal scopa. It can easily be recognized by the following combination of characters: inner metatibial spur with few, elongate branches (Fig. 5); scutum uniformly punctate, with coarse punctures separated by a puncture width or less (Fig. 3); metasomal terga largely impunctate, with minute, faint, scattered punctures, without integumental or apical hair bands (Fig. 4); body pubescence ferruginous; and tibial scopa with sparse, long (2.5–3.0 times median ocellar diameter), apically branched hairs. Among species of the *neotropicus* group, *Leioproctus rosellae* resembles those having an inner metatibial spur with few (8 or less), elongate branches such as the Argentinean species *Leioproctus neotropicus* (Friese) and *Leioproctus stilborhinus* (Moure). However, those species have a different combination of characters, have the clypeus and scutum sparsely punctate, and the metasomal terga more coarsely and densely punctate than in *Leioproctus rosellae*.

#### Description.

 *Female*: Body length 6.56 mm; forewing length 5.0 mm; head width 2.23 mm. Head 1.2× wider than long; inner orbits of compound eyes slightly converging below (Fig. 2); intertorular distance 1.6 times median ocellar diameter, 1.2 times length of torulorbital distance; torulus diameter subequal to median ocellar diameter; ocellocular distance 2.5 times median ocellar diameter, 1.8 times greater than ocelloccipital distance; interocellar distance subequal to ocellocular distance, about 2.4 times median ocellar diameter; compound eye about twice as long as broad; clypeus about twice as broad as long, flat in profile view; gena 0.8 times narrower than compound eye in profile; supraclypeal area gently convex; frontal line distinct, carinate just above inferior torular margin to one-half distance between upper torular margin and median ocellus, ending at that point; facial fovea absent; scape 4.1 times longer than broad; antennal flagellum about twice as long as scape; pedicel subequal to first flagellomere, slightly longer than broad, first flagellomere 1.2 times longer than broad, about twice as long as F2 and F3 individually, remaining flagellomeres broader than long, except last flagellomere longer than broad; glossal lobes broader than long; labial palpus four-segmented; maxillary palpus six-segmented. Propodeum with subhorizontal basal area about as long as metanotum, marginal groove continuous, not pitted; protibial spur with apical portion of rachis long, about half of malus length, with distinct row of 5 elongate branches (not including apical portion of rachis); basitibial plate with apex rounded; mesotibial spur gently curved apically, ciliate, more than one-half of mesobasitarsus length; inner metatibial spur straight, pectinate (Fig. 5), with distinct row of 7 elongate branches on left spur, 5 on right spur (not including apical portion of rachis); pretarsal claws cleft, inner ramus shorter than the outer; arolia present in all legs; forewing with basal vein distal to cu-v.

Color black, except outer surface of mandible and anterior surface of antennal flagellum yellowish and the following areas light to dark reddish brown: antennal scape, tegula, legs excluding coxae and trochanters, and metasoma. Wing membranes brownish, veins and pterostigma dark brown.

**Figures 1–5.**
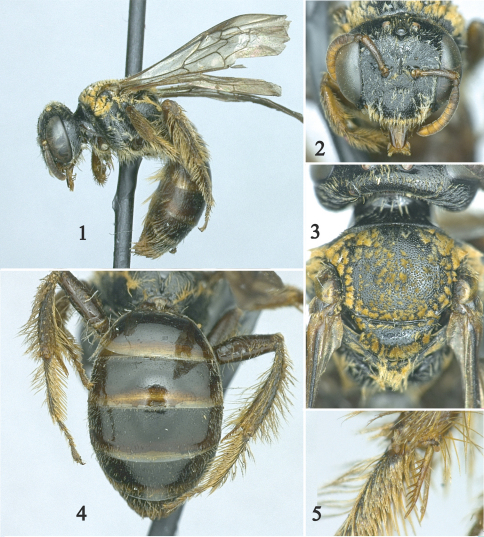
Female holotype of *Leioproctus (Perditomorpha) rosellae* Gonzalez, sp. n. **1** Lateral habitus. **2 **Frontal view **3** Detail of scutum and scutellum **4** Detail of metasoma in dorsal view **5** Left metatibial spurs.

Pubescence light ferruginous, whitish on face. Head with short (less than median ocellar diameter), sparse, plumose hairs except long (≤ 2 times median ocellar diameter), simple, stout hairs on preapical margin of clypeus. Pronotal lobe, mesepisternum dorsally, scutum, scutellum, and metanotum with strongly plumose hairs partially obscuring integument; hairs long (≥ 2 times median ocellar diameter) on scutellum, metanotum, posterior surface of propodeum, and mesepisternum ventrally; femoral and tibial scopa with sparse, long (2.5–3.0 times median ocellar diameter), apically branched hairs (cf. Michener 2007; fig. 13-1a); inner surface of metatibia with simple, shorter hairs than on scopa. Metasomal terga mostly bare, with scattered, minute (~0.2 times median ocellar diameter), erect simple hairs on discs, hairs becoming longer, denser and branched towards apical terga, longer and denser on fifth and sixth terga (Figs 1, 4); second to fifth metasomal sterna distally with long (about 3 times median ocellar diameter), poorly branched hairs.

Integument in general smooth and shiny between punctures, weakly imbricate on metasomal sterna. Outer surface of mandible with sparse, minute punctures; clypeus with larger punctures than on mandible separated by 1–2 times a puncture width; supraclypeal area with sparser punctures than on clypeus; subantennal area and inferior paraocular area with punctures separated by a puncture width; frons with small punctures separated by 1–2 times a puncture width, punctures becoming smaller towards interocellar area; vertex with coarse punctures separated by a puncture width or less; gena with faint, small punctures separated by more than two times a puncture width. Scutum uniformly punctate, punctures coarser than on vertex, separated by a puncture width or less; scutellum sparsely punctate on disc; axilla with small punctures separated by a puncture width or less (Fig. 3); metanotum with large punctures as on scutum separated by a puncture width or less; mesepisternum with punctures separated by more than two times a puncture width; metepisternum minutely punctate. Propodeum impunctate basally, posterior surface with coarse, scattered punctures, punctures becoming sparser and faint on lateral surface. Metasomal terga largely impunctate, with minute, faint, scattered punctures on discs, punctures coarser and denser on fifth tergum; distal margins of terga shiny, smooth and impunctate except on apical terga; sterna with coarse, scattered punctures.

*Male*: Unknown.

#### Etymology.

 This species is named after my newly born daughter Rosella Amparo Betancourt, who was born July 28, 2011, and has already brought us immeasurable love and joy.

#### Comments.

 The holotype is in somewhat poor condition. The left foreleg is missing, the distal margins of the wings are worn out, and the hairs are plastered against the integument (Fig. 1). It is likely that *Leioproctus rosellae* also occurs along the valleys of the Magdalena and Cauca rivers where tropical dry forests occur. Such a distribution pattern is exhibited by some solitary (e.g., *Anthidium sanguinicaudum* Schwarz) as well as social bees [e.g., *Melipona favosa* (Fabricius), *Frieseomelitta paupera* (Provancher)] that also inhabit the same type of forests (Gonzalez, personal observations).

## Supplementary Material

XML Treatment for Leioproctus (Perditomorpha) rosellae
